# Psychodynamic phenomena in obese patients

**DOI:** 10.1192/j.eurpsy.2021.1857

**Published:** 2021-08-13

**Authors:** F. Mustač, S. Bjedov, M. Matovinović, N. Jaksic, B. Vuksan-Ćusa, D. Marčinko

**Affiliations:** Department Of Psychiatry And Psychological Medicine, University Hospital Centre Zagreb, Zagreb, Croatia

**Keywords:** obesity, shame, pathological narcissism, eating

## Abstract

**Introduction:**

Obesity is one of the leading problems of today’s society. According to WHO, 650 million people worldwide are obese, which is 13% of total population (in Croatia 21.5%). There are various psychodynamic theories that interpret the psychological aspects of obesity.

**Objectives:**

The aim of this paper is to present psychodynamic and contemporary psychiatric concepts that explain the interrelated phenomena presenting in obese patients.

**Methods:**

The review of the literature included the investigation of the existing studies in the field of modern psychiatry, as well as previous knowledge in the field of psychodynamics.

**Results:**

Obesity is associated with the emptiness of not recognizing one’s own emotions from hunger, and the need for constant replacement. The everyday life of the obese is filled with shame, an uncomfortable perception that is so intense that can be unbearable. The emptiness and shame which overwhelm and create discomfort cannot be fulfilled by constant food intake and are associated with pathological narcissism (grandiose or vulnerable), which in turn is associated with more regressive behaviour. Thus, obesity may sometimes be associated with addictive behaviours, and a cognition that a bad pattern of rewarding behaviour through food has been adopted in parallel with poor self-control.

**Conclusions:**

Relationship between psychodynamic phenomena and obesity is complex and multidimensional. Further research is needed in order to ameliorate our understanding of these connections.

**Disclosure:**

No significant relationships.

